# Development and Functionality of a Parsimonious Digital Food Frequency Questionnaire for a Clinical Intervention among an Indigenous Population

**DOI:** 10.3390/nu15235012

**Published:** 2023-12-04

**Authors:** Kathleen Abu-Saad, Moran Accos, Arnona Ziv, Fiona Collins, Carrington Shepherd, Sandra Eades, Ofra Kalter-Leibovici

**Affiliations:** 1 Gertner Institute for Epidemiology and Health Policy Research, Sheba Medical Center, Ramat Gan 52126, Israel; 2 South West Aboriginal Medical Service, Bunbury, WA 6230, Australia; 3 Curtin Medical School, Faculty of Health Sciences, Curtin University, Perth, WA 6102, Australia; carrington.shepherd@curtin.edu.au; 4 Melbourne School of Population and Global Health, The University of Melbourne, Melbourne, VIC 3010, Australia; 5 Epidemiology & Preventive Medicine Department, School of Public Health, Faculty of Medicine, Tel Aviv University, Tel Aviv 69978, Israel

**Keywords:** food frequency questionnaire, digital dietary assessment tools, Indigenous populations, Australian Aboriginal adults, cultural adaptation

## Abstract

Nutrition-related chronic diseases are a major problem among Indigenous populations. Appropriate dietary intake assessment tools are needed for nutritional surveillance and intervention; however, tools designed to measure the habitual dietary intake of Indigenous persons are largely lacking. We developed a digital food frequency questionnaire (FFQ) to measure habitual consumption among Australian Aboriginal adults and support personalized nutrition counseling. The primary contributors to energy, select nutrients, and inter-person variation (83 food groups) were identified from nationally representative 24 h recall (24HR) data, and they accounted for >80% of the total intake and inter-person variation of the nutrients of interest. Based on community input, a meal-based FFQ format was adopted, with a main food/beverage list of 81 items and the capacity to report on >300 additional items via the digital platform. The nutrient database was based on the Australian Food and Nutrient Database. Data for the first 60 study participants (70% female; median age: 48 years) were used to assess the FFQ’s utility. The participants’ median [IQR] reported energy intake (10,042 [6968–12,175] kJ/day) was similar to their median [IQR] estimated energy expenditure (10,197 [8636–11,551] kJ/day). Foods/beverages on the main FFQ list accounted for between 66% and 90% of the participants’ reported energy and nutrient intakes; the remainder came from participant-selected extra items. The digital FFQ platform provides a potentially valuable resource for monitoring habitual dietary intake among Aboriginal adults and supporting chronic disease prevention and management interventions.

## 1. Introduction

Nutrition-related chronic diseases (NCDs) are a major and expanding global health problem. This problem is particularly acute among Indigenous populations, which have experienced a loss of traditional lands, lifestyles, and food and social systems as a result of colonization [1,2,3,4,5]. The disruption of traditional food systems has led to the rapid and extensive Westernization of Indigenous food environments and dietary patterns, particularly in urban settings, and consequently, to disproportionately high NCD morbidity and mortality compared to their non-Indigenous counterparts [1,2,3,4,5,6,7,8]. Furthermore, as a result of colonization and ongoing marginalization, Indigenous peoples often suffer from disproportionally high rates of poverty and food insecurity [5,7,8,9,10,11,12]. For the Aboriginal and Torres Strait Islander populations of Australia (hereafter respectfully referred to as Aboriginal), colonization resulted in a gradual shift from a diversified, nutrient-dense traditional diet that was high in dietary fiber and low in refined carbohydrates and fat to an energy-dense, nutrient-poor Westernized diet, high in refined sugars and fat [7]. This transition occurred among all Aboriginal populations. However, there are indications that it has been greater among those living in urbanized/non-remote areas, who reported consuming a higher proportion of total energy from nutrient-poor, energy-dense discretionary foods (42.8%) than their counterparts living in remote areas (35.8%) [8,13].

Diet represents a modifiable risk factor for the prevention and management of NCDs, as well as for reducing NCD-related health disparities. Appropriate dietary intake assessment tools are needed to provide critical evidence for policymaking and intervention [14]. Nutritional surveillance is conducted in many countries using short-term dietary assessment methods such as 24 h recall (24HR) surveys, which elicit a detailed description of dietary intake in the previous 24 h [15] and have been used as a basis for estimating the adequacy of dietary intake at a population level. However, since such short-term assessment methods do not capture day-to-day variation in intake, they are not suitable for estimating an individual’s long-term habitual dietary intake, which is related to the development of NCDs and their sequelae [16].

The primary method used for estimating habitual dietary intake in epidemiological studies of diet–chronic disease associations is the food frequency questionnaire (FFQ), which elicits data on the usual intake frequency and quantity of a list of foods commonly consumed [17]. Food intake varies across populations and their subgroups; thus, to accurately capture dietary intake, the food list must be appropriate for the study population [17,18]. Numerous studies have demonstrated that foods making important contributions to the energy and nutrient intakes of specific minority groups are missing from standard FFQs developed for their respective general populations (e.g., Hispanics [19] and Caribbean-origin Blacks [20] in the U.S.; Bedouin Arabs in Israel [21]; Surinamese, Turks and Moroccans in the Netherlands [22]). An FFQ developed for and validated in a particular society cannot be assumed to be a valid instrument for use in all of its subgroups, and often Indigenous populations are not adequately represented in the development of national FFQs [23]. An FFQ developed for and validated among Australian adults had poorer performance among Southern European (Greek/Italian) immigrants than among Australian-born adults. Aboriginal Australians were not included in this study at all [23], nor in any of the other FFQs that were developed and validated for use in Australia [24,25,26,27]. A 2017 literature review [7] identified only one FFQ developed for an Aboriginal population—a short, 28-question instrument for rural Indigenous and non-Indigenous children aged 10–12 years [25]. There is, in general, a lack of current, representative data on the dietary intake of Aboriginal Australians, with the exception of the Australian National Aboriginal and Torres Strait Islander Nutrition and Physical Activity Survey (NATSINPAS) conducted by the Australian Bureau of Statistics (ABS) from 2012–2013 [7,28], which collected 24HR data. Reports published by the ABS indicated that the intakes of numerous nutrients and food groups among Aboriginal Australians differed from those of non-Aboriginal Australians, including lower fruit, vegetable, whole grain and dairy intakes, and higher discretionary food and saturated fat intakes [13,29]. These summary documents, however, did not provide a comprehensive, granular report of the items that accounted for these differences, so the precise ways in which the food and beverage intake of Aboriginal people differs from non-Aboriginal people is not known.

It is also important that the dietary assessment tools used in clinical interventions and practice be appropriately adapted for the clinical aims and constraints [7,17,20,30]. In this article, we describe the development of an FFQ for use in the Kaat Koort Aboriginal Brain Health Study among Aboriginal adults in selected urban and regional areas of Western Australia. The Kaat Koort study is a multimodal intervention, tested in a randomized controlled trial, that was designed to reduce the risk of dementia and cognitive decline by better controlling cardiovascular risk factors (e.g., hypertension, hyperlipidemia) and optimizing lifestyle behaviors (e.g., diet, physical activity, smoking) [31,32]. The study protocol included assessing habitual dietary intake and providing individualized dietary counseling to increase adherence to the Australian Dietary Guidelines (ADGs) [33] over the course of a 12-month intervention. Given that no FFQ had been designed or validated for Australian Aboriginals, we utilized the NATSINPAS 24HR data to create a data-based FFQ using a customizable Interactive Lifestyle Assessment, Counseling and Education (I-ACE) digital platform that was previously piloted among another marginalized Indigenous group [34,35]. The clinical aims and constraints of the Kaat Koort study further required that the FFQ provide as comprehensive a picture of the total habitual intake as possible, balanced against the need for brevity to enable its use in a clinical setting. In this article, we describe the development of the digital FFQ to meet these specifications and a pilot assessment of its functionality.

## 2. Materials and Methods

### 2.1. FFQ Development

**Figure 1:**
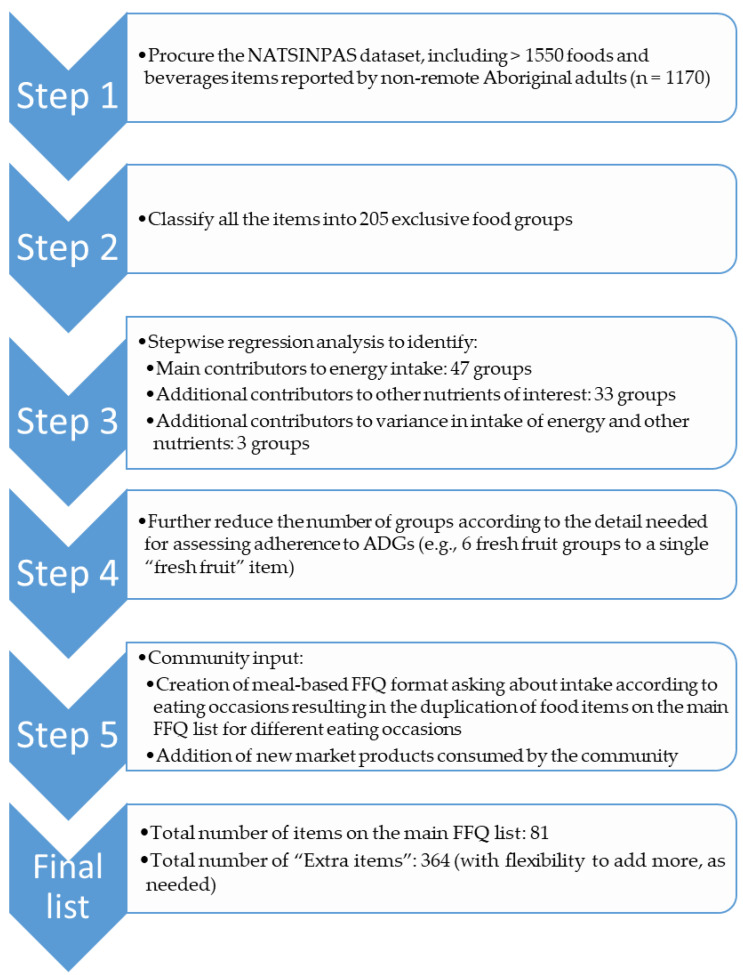
Flowchart of the development of the food frequency questionnaire (FFQ) for use among non-remote Aboriginal adults in Australia. ADGs, Australian Dietary Guidelines; NATSINPAS, National Aboriginal and Torres Strait Islander Nutrition and Physical Activity Survey (conducted from 2012–2013).

The FFQ was developed for Australian Aboriginal adults living in non-remote settings via a 5-step process (see Figure 1). This included initial identification of food and beverage items for the FFQ list using a population-specific, large-scale, representative dietary intake data source, supplemented with guidance from community members.

#### 2.1.1. Construction of an Appropriate Food List

- Step 1: Access to population-specific dietary intake data. The Australian National Aboriginal and Torres Strait Islander Nutrition and Physical Activity Survey (NATSINPAS) was identified as the primary candidate for population-specific dietary data to inform the development of the FFQ. It included a representative sample of 2300 non-remote and 1800 remote Aboriginal and Torres Strait Islander participants aged ≥ 2 years from 2900 households (one adult and one child per household). The survey was conducted by the Australian Bureau of Statistics (ABS) in 2012–2013 and had a 79% response rate. The ABS published a detailed description of the sampling framework and data collection methodology [28].
        The NATSINPAS dietary intake data were collected in person by trained interviewers using the US Department of Agriculture’s (USDA’s) Automated Multiple-Pass Method 24HR questionnaire [36,37], adapted to reflect the Australian food supply [38]. A second 24HR was collected by phone in a subsample of volunteer participants. We used the basic Confidentialized Unit Record Files (CURF) data for non-remote participants aged ≥ 18 years (n = 1170) to guide the development of the FFQ food list for the Kaat Koort study. Only data from the first 24HR were used in these analyses because the second 24HR was completed by a self-selected subsample of participants and there is no published information about the response rate or representativeness of this subsample [29].
- Step 2: Data classification. In the ABS dataset, all the foods reported in the 24HR data were classified into major, sub-major and minor food groups, based on the US National Health and Nutrition Examination Survey (NHANES) classification system [39]. For the purposes of creating the FFQ, the >1550 food and beverage items reported on the 24HRs were classified into 205 food groups (Supplemental File S1—Table S1).

- Step 3: Data analysis. These food groups were entered into a stepwise multiple regression model according to their specific nutrient content and their intake quantity from the 24HR [40]. This procedure was conducted for energy and selected nutrients that either determine energy intake or have been associated with cardiovascular health (protein, fat, carbohydrates, fiber, sodium, potassium and magnesium) [41]. To ensure an adequately comprehensive FFQ food list, food groups that accounted for at least 80% of the total intake of these nutrients were considered for inclusion in the FFQ food list. To simultaneously maximize the capacity of the FFQ to rank participants according to nutrient intake levels, the food groups that explained at least 80% of the between-person variability for each of the nutrients of interest were also identified and considered for inclusion in the FFQ. To ensure participants were not overburdened, we aimed to develop a list of less than 100 food and beverage items to be assessed among all participants. Additional items reported by NATSINPAS participants were retained in the food database. They were assigned to the appropriate food group and included in the digital I-ACE FFQ platform as “Extra items” that could be selected and added to the FFQ during the assessment process by participants who consumed them.

#### 2.1.2. Refining the List

**Figure 2:**
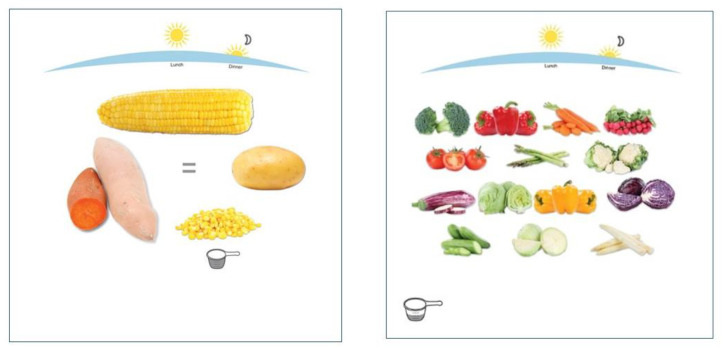
I-ACE platform food pictures for “Starchy vegetables” and “All other vegetables”.

- Step 4: Abbreviation of list items. Since the overall aim of the dietary counseling was to improve adherence to the food-based ADGs [33], this guided the abbreviation of the FFQ food list. For example, starchy vegetables (potatoes, corn, sweet potatoes) were grouped into a single item, as were fresh vegetables. The electronic platform included pictures to inform the user of the foods each FFQ item included (see Figure 2).
- Step 5: Community input. Critically, we obtained community input to refine the data-derived food list and database. An experienced local dietician working with the South West Aboriginal Medical Service in Bunbury, Western Australia (one of the two study sites) assisted with the prioritization of foods to be included in the main FFQ list. She also reviewed and assisted with: (1) defining serving sizes as amounts typically served/eaten/packaged (e.g., 1 slice of bread, 1 flatbread); (2) defining ADG-based serving sizes (e.g., 1 ADG grain serve = 1 slice of bread, ½ flatbread); and (3) reviewing the food database for completeness.

The consultant dietician suggested that a meal-based FFQ be used rather than the standard FFQ food list format that requires respondents to recall and sum their intake of an item over all consumption occasions (e.g., breakfast, lunch, dinner, between-meal snacks). We thus formatted a meal-based FFQ to create a more culturally congruent “yarning” dynamic, utilizing a conversational process to report dietary intake in the context of meals throughout the day. The term “yarning” is commonly used by Aboriginal people to describe a conversation, where information is exchanged in an informal way. It is a popular method employed in gathering information in qualitative research that aligns with Indigenous ways of sharing knowledge, is culturally safe, and involves a two-way process that is mutual or reciprocal [42,43,44,45]. The dieticians first asked participants about the foods they usually ate at breakfast from the FFQ Breakfast list of bread, spreads, dairy, eggs, meat, vegetables, etc. (or “Extra items” lists), including how often and how much they consumed of each reported item; second, they asked about the foods on the FFQ Lunch/Dinner list (or “Extra items” lists) that they ate at lunch and dinner; and third, they asked about the foods on the FFQ Snacks list they ate as between-meal snacks. Beverages and supplements (e.g., vitamins, minerals, protein, fiber) were separate categories, for which respondents were asked to sum their intake over all consumption occasions throughout the day. Both the standard and meal-based formats were piloted among Aboriginal health professionals (AHPs) working at the clinic. The observed discrepancies in reporting between the two FFQ formats were reviewed with the pilot participants, and they confirmed that the reports in the meal-based FFQ produced a more accurate reflection of their intake (e.g., the total average daily amount of bread reported was greater with the meal-based than the standard FFQ, and the pilot participants confirmed that the amount reported with the meal-based FFQ was more accurate and not the result of over-reporting). Since the meal-based format increased the FFQ administration time by only 10–15 min, it was selected for the study FFQ format.

### 2.2. Developing a Study Food and Nutrient Database

Foods and beverages reported in the NATSINPAS data were linked to nutrient data from the Australian Food and Nutrient Database (AUSNUT), which was developed and customized by Food Standards Australia New Zealand and included 44 nutrients [37,38,39]. The nutrient values in the study food and nutrient database were derived from the AUSNUT nutrient values for the individual food items that NATSINPAS participants reported consuming, weighted by their consumption quantity. For example, for the FFQ item “Fresh/frozen fruit”, 35 discrete fruit items were reported in the NATSINPAS 24HR data. The intake in grams for each item was summed and divided by the total grams for all fruit items to derive a proportional weight for each fruit item. These weights were then used to calculate the nutrient values for the composite “Fresh/frozen fruit” FFQ item. This process is illustrated in Supplemental File S1—Table S2.

**Figure 3:**
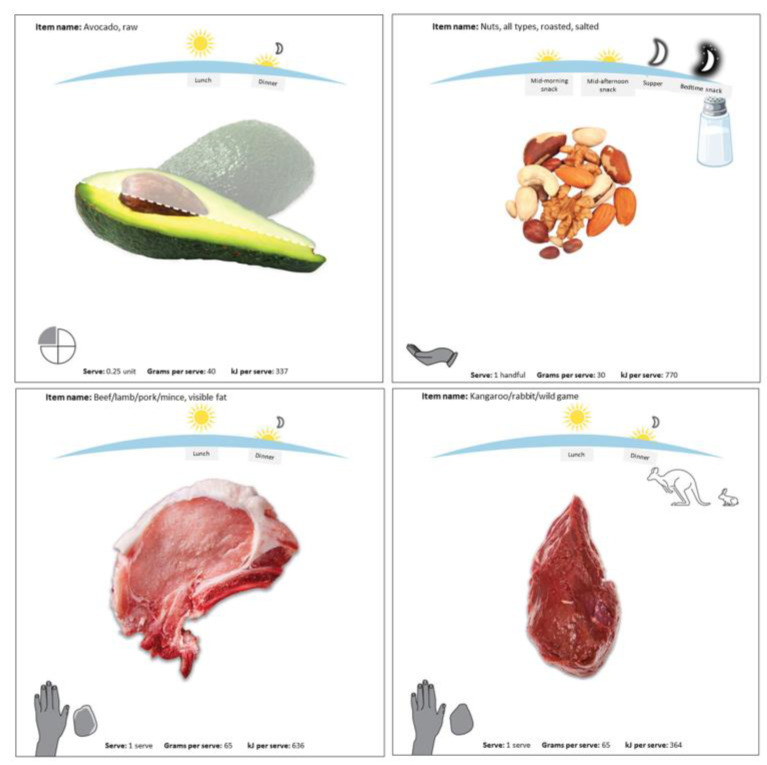
I-ACE platform digital reporting aids illustrating eating occasion, food, and portion size.

A serving size was set for each item, based on natural units to facilitate reporting (e.g., 1 egg, 1 hotdog in a bun), and was illustrated with digital aids, including pictures, portion icons (e.g., unit, cup, tablespoon, handful, fist- or palm-sized portion), grams and kilojoules (kJs) of the serving size in the I-ACE platform (Figure 3). Where applicable, items were also classified according to standard ADG servings, as defined in the AUSNUT database and ADG guidelines (e.g., 1 egg = 0.5 ADG Meat and Alternatives servings) [33]. For discretionary foods (e.g., sweets, sugar-sweetened soft drinks, processed meats, commercial burgers and fried foods, salty snacks), we used the ADG definition of 500–600 kJs as a discretionary serving [33] to calculate the number of discretionary servings in a portion (e.g., 1 hot dog in a bun = 1443 kJ = 2.6 discretionary servings).

The study dieticians and AHPs also suggested the addition of new foods/beverages that came on the market after the NATSINPAS was conducted. Since the study FFQ served as a basis for nutritional counseling, additional items that dieticians could suggest as healthier options were added to the “Extra items” lists (e.g., reduced/low fat dairy products, healthier fast food options such as fresh vegetable sides, healthy traditional foods such as kangaroo/wild game). We took the nutrient values for these new items from AUSNUT if available; and if not, from other sources, such as Calorie King (https://www.calorieking.com/au/en/ (accessed on 30 November 2023)) or product websites.

### 2.3. Assessing FFQ Functionality

To provide some initial metrics on the functionality of the FFQ, we analyzed the baseline dietary intake data of the first 60 participants in the Kaat Koort study who completed the FFQ.

#### 2.3.1. Participant Recruitment

Study participants were recruited from the South West Aboriginal Medical Service in Bunbury (beginning in August 2021) and from the Derbarl Yerrigan Health Service in Perth (beginning in July 2022). Aboriginal people in the study communities who had cardiovascular risk factors and/or atherosclerotic cardiovascular disease (ACVD) and were able to participate in a lifestyle intervention were recruited.

#### 2.3.2. FFQ Administration

**Figure 4:**
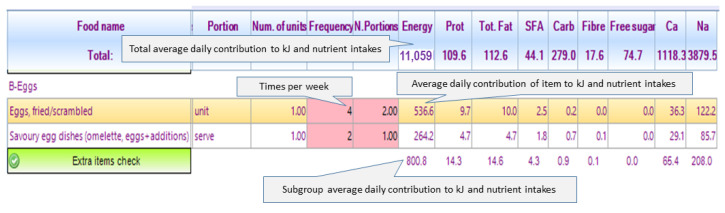
Excerpt of the food frequency questionnaire on the I-ACE platform.

After undergoing training in using the I-ACE software (v3.0) for dietary assessment and counseling, the study dieticians administered the FFQ to participants in one-on-one, in-person sessions. The dieticians asked participants to think about the previous month and report their usual intake (frequency and number of portions) of the items on the meal-based list of food subgroups (e.g., Breakfast bread, spreads, dairy). The platform required the dieticians to provide consumption data for all of the items on the main food list and to check the “Extra items” list of each food subgroup, either indicating that no other foods were reported or adding reported foods to the FFQ and providing consumption information (see Supplemental File S3 for a screenshot of the digital FFQ with explanation of the “Extra items” lists usage). As the consumption data were entered, the software automatically calculated the contribution of the food item to the participant’s average daily energy and nutrient intakes, as well as to relevant ADG food groups (Figure 4).

Ethical approval was received from the Western Australian Aboriginal Health Ethics Committee (947) and the University of Melbourne Medicine and Dentistry Human Ethics Subcommittee (2056720).

## 3. Results

### 3.1. Analysis of NATSIPAS Nutrition Data and Development of the FFQ

Forty-seven (47) of the 205 food groups reported in the NATSIPAS data accounted for 80% of the total energy intake reported by non-remote Aboriginal NATSIPAS participants (Table 1) in the 24HR. To account for the intake of 80% of the nutrients of interest, we identified 14 additional food group contributors to the macronutrients (protein, total fat, carbohydrates) and 19 additional food group contributors to fiber, sodium, potassium and magnesium (Table 1; see also Supplemental File S4 for a more detailed list). The regression analyses identified another three food groups that were not among the top contributors to total intake but contributed to explaining 80% of the between-person variance in the intake of energy and the nutrients of interest (Table 1).

The identified food groups were candidates for inclusion in the study FFQ. Given our aim of measuring changes in adherence to the ADG goals while keeping the length of the FFQ feasible for use in a clinical intervention, we combined selected food groups. For example, the six fresh fruit groups (bananas, pome, citrus, stone, tropical fruit, and other fruits) were combined into one item in the main FFQ list (“Fresh/frozen fruit”). Less commonly consumed items within a food group were included in an “Extra items” list for each food group on the main FFQ list.

After additional input from the consultant and study dieticians and AHPs, the main FFQ food list consisted of 81 items, with another 364 items on the “Extra items” lists (Figure 1). These numbers included the repetition of items so that they could be reported according to the eating occasion. Eight foods (e.g., sausage, fried/scrambled eggs, some fast foods) were repeated in two meal occasions, and seven foods were repeated in three meal occasions (e.g., white flour and whole meal bread, butter, margarine, starchy vegetables).

Table 2 presents the five highest 24HR food group sources of energy with the food and beverage items used to represent them in the digital FFQ platform.

### 3.2. Test of the FFQ among Kaat Koort Participants

Data from the baseline FFQ assessments of 60 participants (collected as of 24 October 2022) were used in these preliminary analyses. Most participants (70.0%) were women, and their median (IQR) age was 48 (42–55) years (Table 3). Consistent with the inclusion criteria for the study that targeted people with cardiovascular risk factors and/or ACVD, 38.3% had hypercholesterolaemia, 21.7% had hypertension, 41.7% had type 2 diabetes, and 11.5% had coronary artery disease. The median (IQR) BMI was 34.0 (29.0–58.4) kg/m^2^. The median (IQR) reported energy intake was 10,042 (6968–12,175) kJ per day. Using the Dietary Reference Intake equation for total energy expenditure (TEE) [46], the estimated median (IQR) TEE was 10,197 (8636–11,551) kJ per day.

Table 4 indicates that the main items on the FFQ list accounted for between 66% and 90% of the Kaat Koort participants’ total reported energy and nutrient intakes (4a), while the remainder came from participant-selected extra items. The variation was greater for food groups, with the items on the main FFQ list accounting for 51–97% of the total reported serves (4b). The main FFQ list items accounted for a higher proportion of the total reported intake if they represented categories (e.g., fresh/frozen fruits, fresh vegetables) rather than 1–2 specific, individual foods (e.g., full-fat milk, regular-fat flavored yogurt), with many other specific items in extra items (e.g., low-fat milk, yogurts, hard/soft/spreadable cheeses). For example, for the dairy group, the main FFQ list had only milk and flavored yogurt in the Breakfast dairy group, and only hard yellow cheese in the Lunch/Dinner and between-meal Snacks dairy groups; while many other types of milk, yogurt and cheese could be selected from the extra items list at each of these eating occasions. Thus, in the case of the total dairy intake, the items on the main FFQ list accounted for only 56.8% of the reported dairy serves, while the items from the “Extra items” accounted for 43.2%. Supplemental File S5 presents these analyses by sex and shows similar tendencies. The results suggest that there may be differences in reporting between men and women; however, the pilot sample was too small to draw reliable conclusions.

Based on these preliminary data, we created an offline version of the FFQ. It included the main FFQ list, with space for writing in extra items at the end of each eating occasion/food subgroup. The most frequently reported extra items for each group were included in parentheses as examples (Supplemental File S6).

## 4. Discussion

This paper describes the development of a digital, data-based FFQ for use in assessing dietary intake associated with CV health and adherence to the ADGs among a group of Indigenous adults in Australia. The FFQ was culturally adapted through the development of the food list and the meals-based format, and it was designed to support personalized nutritional counseling and follow-up as part of the lifestyle intervention in the Kaat Koort Aboriginal Brain Health Study. Beyond the current study, it may offer a potential resource for providing dietary assessment and advice to improve cardiovascular health in clinical settings among non-remote Aboriginal Australian adults and a model for developing appropriate FFQs for other populations.

### 4.1. FFQ List Development

The dietary assessment methodology literature identifies the construction of an appropriate food list as the most critical part of FFQ development. If important items are omitted, energy and nutrient intakes may be underestimated, while if too many irrelevant items are included, the participant burden is unnecessarily increased [17]. Representative data on total dietary intake in the target population provide a basis for developing an appropriate FFQ food list and have been used extensively [17,47,48,49,50,51,52,53], including for Indigenous populations [17]. We used data from a population-based dietary assessment of Australian Aboriginal adults and the method described by Willett [30] to identify the key list of foods and beverages to consider for inclusion in the FFQ. We then abbreviated the list based on our research interests in (a) improving cardiovascular health, (b) measuring adherence to the food-based (rather than nutrient-based) ADGs, (c) including the input of local dieticians and AHPs, and (d) constructing a relatively short FFQ that could be used in a clinical intervention. Our decision, for example, to include all fruits in two items (fresh/frozen fruit, dried fruit) enabled assessment of ADG adherence but would not provide precise estimations of the intake of specific micronutrients. Researchers whose aims require more precise assessments of certain micronutrients can replicate the data reduction methods from the raw 24HR data and include the foods that are important to estimating the intake of these micronutrients.

### 4.2. Community Input and Meal-Based FFQ

In addition to constructing an appropriate FFQ food list, we followed community-based input to adapt the FFQ format from the classic food list to a meal-based list and administered it with a two-way, reciprocal conversational approach. This adaptation coincides with Indigenous methodologies for contextualized, holistic research and knowledge generation [42,43,44,45,54]. There are limited examples of meal-based FFQs in the literature, although studies show that they exhibit correlations similar to those of standard FFQs to reference methods (e.g., 24HR, weighed food records, doubly labeled water) [55,56,57]. Depending upon factors such as how they were administered (period to be covered) and length of the food list, meal-based FFQs either underestimated [55] or overestimated [57] energy intake. However, Quandt et al. [57] found that the meal-based FFQ had stronger correlations with the average of multiple 24HRs than the standard FFQ among women and similar correlations to the standard FFQ among men. Existing meal-based FFQ studies focused on the ranking ability of the FFQ for epidemiological research; none were used in clinical dietary counseling contexts. Although one was web-based [55], it appeared to have a limited food list, without the capacity for participants to report on additional foods, and the median energy intake estimate (7667 kJ) was substantially lower than that of the weighed food records (9183 kJ) and doubly labeled water (11,423 kJ). In addition, it did not utilize food pictures.

### 4.3. Advantages of the Digital Platform

In contrast to Christiansen et al.’s [55] results, the Kaat Koort participants’ median reported energy intake (10,042 kJ) was quite close to their median estimated total energy expenditure (10,197 kJ), despite the fact that the main FFQ food list was relatively short (81 items). This highlights the advantage of the digital platform we used, which enabled individuals to easily report foods less commonly consumed on a population-wide basis. Originally, paper FFQs typically had extra lines for participants to write in items they consumed that were not on the FFQ list [58,59,60,61]. The digital FFQ platform maintains and enhances this capacity, not only by allowing for the addition of foods not on the main FFQ list but also by sorting and displaying extra items by food group, which may serve to remind respondents of additional items they consume. As such, this dietary assessment tool represents a pioneering effort to more fully exploit the advantages of a digital platform to create an FFQ that is concise enough for use in an intervention setting, while preserving the capacity to support comprehensive dietary intake reporting.

### 4.4. Strengths and Limitations

This FFQ was developed within the context of a research project that did not have designated funding for validating the FFQ via additional reference methods (e.g., multiple 24HRs, weighed food records); thus the purpose of this article was to describe the FFQ development process and present preliminary data on its functionality. Additional studies should be designed and conducted to validate the FFQ for the non-remote Aboriginal adult population. Nevertheless, we note that the median reported energy intake was close to estimated total energy expenditure. The capacity of the digital FFQ platform to incorporate extra items not on the main food list provides some of the advantages of the reference validation methods mentioned above by facilitating the reporting of all items a respondent regularly consumes rather than being limited to, or reminded only of, a closed list of items. The data-derived FFQ was developed for a specific Indigenous population (non-remote Aboriginal adults) and is not necessarily appropriate for other Indigenous groups, which exhibit vast diversity within and between countries. However, the data analysis process can be replicated for other groups using population-specific data to create relevant and appropriate FFQ food lists.

The study strengths include the use of large, representative survey data to develop, to the best of our knowledge, the first FFQ specifically designed for the non-remote adult Aboriginal population of Australia. Its development was further actively guided by community-based input.

## 5. Conclusions

This study describes the model and methods for developing an FFQ for a specific Indigenous population and defined clinical intervention aims. The FFQ and digital platform provide potentially valuable resources for monitoring habitual dietary intake and adherence to dietary recommendations in this community. The methodology provides a model for FFQ development that can be replicated for other Indigenous/minoritized populations to support interventions aimed at chronic disease prevention and management.

## Figures and Tables

**Table 1:** Food groups identified from NASTINPAS 24HR data for possible inclusion in the Kaat Koort Aboriginal Brain Health Study FFQ by percent contribution to total energy intake.

Food Groups	Percent Contribution to Total Energy Intake
Main contributors to energy intake ^1^	
Breads, white	6.45
Beef, lamb, pork	4.70
Soft drinks	3.75
Processed meat	3.47
Hot potato chips	3.40
Milk, full fat	3.13
Mixed poultry dishes	3.08
Savory pasta/rice dishes	3.06
Cakes	2.71
Poultry	2.69
Burgers	2.63
Savory pastry	2.52
Sugar	2.18
Mixed red meat dishes	2.17
Beers, regular	2.01
Pizza	1.79
Wine	1.66
Cheese, ripened, high fat	1.61
Beers, lite	1.54
Potatoes	1.49
Fin fish, fried	1.32
Breads, whole grain	1.27
Breads, mixed grain	1.21
Ice cream, full fat	1.19
Breakfast cereal, whole grain, low sugar	1.17
Cordials	1.15
Chocolate, filled	1.07
Rice	1.02
Margarine	0.97
Eggs	0.94
Milk drinks, full fat	0.92
Bananas	0.92
Butter	0.89
Breakfast cereal, muesli	0.83
Sweet biscuits, filled	0.82
Spiked soft drinks	0.79
Chocolate, plain	0.78
Milk, low fat	0.78
Fruit drinks	0.75
Sandwiches	0.74
Potato crisps	0.74
Porridge	0.72
Pasta and noodles	0.69
Fruit juices	0.68
Pome fruit	0.66
Egg dishes, savory	0.60
Coffee with milk	0.60
*Subtotal % (number of groups)*	*80.25 (47)*
Main contributors to other nutrients ^2^	
Cocktails	0.59
Gravies	0.58
Electrolyte drinks (sports drinks)	0.56
Other nuts	0.53
Salad dressing, full fat	0.53
Doughnut/crepe/pancake	0.47
Dairy desserts	0.47
Salads, vegetable based	0.47
Squash	0.40
Candies, sugar sweetened	0.36
Fin fish, fresh	0.34
Soup with meat, homemade	0.34
Fin fish, preserved	0.33
Mixed vegetables	0.30
Fortified beverage	0.29
Other fruit	0.29
Honey and sugar syrups	0.29
Sports/protein beverage	0.27
Citrus fruit	0.26
Legume products	0.20
Other root vegetables	0.19
Sweetcorn	0.18
Stone fruit	0.17
Carrots	0.15
Tomato	0.15
Peas and edible podded peas	0.10
Brassica vegetables	0.07
*Subtotal % (number of groups)*	*8.88 (27)*
Additional contributors to between-person variation ^3^	
Peanut products	0.23
Spirits/liquors	0.25
Tropical fruit	0.11
*Subtotal % (number of groups)*	*0.59 (3)*
Total contribution of all food groups	89.72 (77)

^1^ Accounted for up to 80% of the total energy intake in the NASTINPAS 24HR data. ^2^ Additional food groups accounting for up to 80% of the total intake of nutrients of interest (protein, total fat, carbohydrates, fiber, sodium, potassium, magnesium) in the NASTINPAS 24HR data. ^3^ Additional food groups identified in the regression analyses as accounting for up to 80% of the between-person variation in energy and nutrients of interest (protein, total fat, carbohydrates, fiber, sodium, potassium, magnesium) in the NASTINPAS 24HR data. Subtotals for each type of contributor are in italic font.

**Table 2:** Five top food group contributors to 24HR energy intake and the food and beverage items representing them in the digital FFQ platform.

24HR Food Groups (% Contribution to Total Energy)	Items Representing Food Group in Digital FFQ Platform
Breads, white (6.4%)	Bread/toast, white flour
	Bread, damper, white flour
	Bread roll, white flour
	Bread, tortilla/flat wrap, white flour
Soft drinks/cordials (4.9%)	Soft drink/fruit drink/cordial/slushie, regular
Beef, lamb, pork (4.7%)	Beef/lamb/pork/mince, visible fat
	Beef/lamb/pork/mince, lean
Processed meat (3.5%)	Bacon
	Sausage
	Sausage, lean
	Ham
	Spam/polony/processed luncheon meat
	Corned beef
	Cheese sausage
Hot potato chips (3.4%)	Hot potato chips/fries/hash browns, takeaway
	Hot potato chips/fries, homemade

**Table 3:** Characteristics of the initial Kaat Koort participants with the study FFQ (n = 60).

Characteristic	Total (n = 60)	Women (n = 42)	Men (n = 18)
Age (years), median (IQR)	48 (42–55)	48.0 (42.0–55.0)	48.5 (42.0–54.0)
Self-reported chronic morbidity			
Hypercholesterolaemia, n (%)	23 (38.3)	16 (38.1)	7 (38.8)
Hypertension, n (%)	13 (21.7)	7 (16.7)	6 (33.3)
Type 2 diabetes, n (%)	25 (41.7)	17 (40.5)	8 (44.4)
Coronary artery disease, n (%)	6 (11.5)	3 (7.1)	3 (16.7)
BMI (kg/m^2^), median (IQR)	34.0 (29.0–58.4)	35.0 (29.3–41.7)	31.7 (28.1–39.8)
Reported energy intake per day (kJ), median (IQR)	10,042 (6968–12,175)	9226 (6645–11,770)	11,200 (8605–13,738)
Estimated total energy expenditure per day (kJ), median (IQR)	10,197 (8636–11,551)	9010 (8223–10,789)	12,413 (10,798–13,564)

**Table 4:** Contributions of the main and “extra” ^1^ I-ACE FFQ items to (4a) the total intake of energy, and selected nutrients and (4b) food groups as reported by initial Kaat Koort participants (n = 60).

	Percent Contribution to Total Intake	
Nutrient or Food Group	Main Items	Extra Items
4a. Energy and nutrients		
Energy (kJ)	69.9	30.1
Protein (g)	66.8	33.2
Total fat (g)	66.0	34.0
Fiber (g)	81.3	18.7
Carbohydrates (g)	74.2	25.8
Total sugar (g)	75.7	24.3
Free sugar (g)	77.3	22.7
Calcium (mg)	65.9	34.1
Sodium (mg)	68.8	31.2
Magnesium (mg)	68.9	31.1
Potassium (mg)	69.9	30.1
4b. Food groups (serves)		
Total grains	79.7	20.3
Whole grains	84.5	15.5
Total Vegetables	90.4	9.6
Fruit	92.3	7.7
Total dairy	56.8	43.2
Low fat dairy	50.7	49.3
Meats and alternative protein sources ^2^	53.8	46.2
Fast/fried food	61.4	38.6
Processed/salty food	61.4	38.6
Alcoholic drinks	69.1	30.9

^1^ Items not on the main food list that were chosen by participants who consumed them from the “Extra items” lists. ^2^ Excluding dairy items (which are in the dairy group) and legumes (which are in the vegetables group).

## Data Availability

The 24 h recall dietary data underlying this article were provided by the Australian Bureau of Statistics (ABS) and cannot be publicly shared by the authors. Researchers wanting to access these data will need to apply to the ABS (https://www.abs.gov.au/statistics/microdata-tablebuilder/microdatadownload) (accessed on 30 November 2023). The data collected from participants in the Kaat Koort study are not publicly available due to the restrictions that have been applied by the approving ethical committees. These primarily concern protecting the privacy and confidentiality of participant data.

## Supplementary Materials

The following supporting information can be downloaded at https://www.mdpi.com/article/10.3390/nu15235012/s1, Table S1: List of the 205 food groups derived from the NATISNPAS dietary intake data of non-remote Aboriginal Australian adults that were entered into regression analyses to identify the main contributors to nutrient intake and variability in intake; Table S2: Total amount in grams of fresh fruit items reported in the NATSINPAS and weights for calculating the nutrient values of the composite fresh/frozen fruit FFQ item; File S3: Screenshot of the digital FFQ with explanation of “Extra item” list usage; File S4: Detailed list of 24HR food and beverage group contributors to kJ and other nutrients of interest; File S5: Contributions of the main and “extra” I-ACE FFQ items to the total intake of energy, selected nutrients and food groups reported by the initial Kaat Koort participants by sex; File S6: Offline Kaat Koort food frequency questionnaire.
