# Supplementary material for: Development and Functionality of a Parsimonious Digital Food Frequency Questionnaire for a Clinical Intervention among an Indigenous Population
Source: Nutrients. 2023 Dec 4;15(23):5012. doi: 10.3390/nu15235012 (PMC10707983; doi:10.3390/nu15235012)
Supplement: Supplementary file 1 [file nutrients-15-05012-s001.zip › Supplemental File 1-2. Tables S1,S2-r2.pdf]

Supplemental File 1-Tables S1, S2

Table S1. List of the 205 food groups derived from the NATISNPAS 24HR dietary intake data of non-remote Aboriginal Australian adults that were entered into regression analyses to identify main contributors to nutrient intake and between-person variability

Group Number	Food group
1	Beef-lamb-pork
2	Processed meat
3	Mixed poultry dishes
4	Poultry
5	Potato products
6	Mixed red-meat dishes
7	Margarine
8	Savory pasta/rice dishes
9	Savory pastry
10	Burgers
11	Milk, full fat
12	Cakes
13	Fin fish, fried
14	Cheese, ripened, high fat
15	Eggs
16	Other nuts
17	Potato crisps
18	Pizza
19	Chocolate, filled
20	Salad dressing, full fat
21	Butter
22	Breads, white
23	Squash
24	Egg dishes, savory
25	Ice cream, full fat
26	Chocolate, plain
27	Peanut products
28	Potatoes
29	Sweet biscuits, filled
30	Salads, vegetable based
31	Margarine, low fat
32	Sandwiches
33	Peanuts
34	Milk drinks, full fat
35	Dairy desserts
36	Pasta and noodles
37	Coffee with milk
38	Soup with meat, homemade

Group Number	Food group
39	Dairy blend, high fat
40	Fin fish, fresh
41	Porridge
42	Sweet biscuits, plain
43	Breakfast cereal, whole grain, high sugar
44	Breads, mixed grain
45	Taco-tortilla
46	Milk, low fat
47	Extruded snacks
48	Mixed fish dishes
49	Savory biscuits, high calorie
50	Batter-based products
51	Salads, vegetable based, added meat, fish or eggs
52	Cream, full fat
53	Breads, whole grain
54	Sweet pastry, filled
55	Mixed processed meat dishes
56	Muesli bars
57	Corn chips
58	Fin fish, preserved
59	Savory sauces, homemade
60	Savory pastry, fried
61	Vegetables and sauce
62	Gravies
63	Oil, olive
64	Shellfish, fried
65	Soup, vegetable, homemade
66	Sweet breads
67	Other root vegetables
68	Yogurt, full fat
69	Carrots
70	Yogurt, flavored, full fat
71	Tomato
72	Savory biscuits, plain, low calorie
73	Fortified beverage
74	Sports/protein beverage
75	Legume based dips
76	Fried vegetable dishes
77	Popcorn
78	Coffee
79	Dairy dips
80	Milk drinks, low fat

Group Number	Food group
81	Onion, leek and garlic
82	Other snacks
83	Egg dishes, sweet
84	Fried bread
85	Frozen dairy desserts, other
86	Breakfast cereal, whole grain, low sugar
87	Sweet spreads-sauces
88	Other breads
89	Meat substitutes
90	Whole grains
91	Mixed vegetables
92	Dairy blend, low fat
93	Pickles
94	Mixed fruit dishes
95	Sweetcorn
96	Nut/seeds, sweet
97	Vegetable-based dips
98	Ice cream, medium fat
99	Soy beverages, plain
100	Pastry, plain
101	Candies, sugar sweetened
102	Kangaroo-mammalian game
103	Liquors
104	Cheese, ripened, low fat
105	Meal replacement beverage
106	Cheese, unripened, full fat
107	Sushi
108	Commercial soups with meat
109	Mushrooms
110	Brassica vegetables
111	Unfortified beverage
112	Cream, sour
113	Custard, low fat
114	Yogurt, flavored, low fat
115	Cream, sour, low fat
116	Wild mammalian meat
117	Mixed dried fruit-nuts
118	Commercial soups, vegetable
119	Seeds
120	Sweet-filled breads
121	Legume products
122	Breakfast cereal

Group Number	Food group
123	Oils PUFA
124	Yeast
125	Shellfish
126	Cheese, unripened, low fat
127	Savory biscuits, whole grain
128	Breads, gluten free
129	Salad dressing, low fat
130	Reptiles
131	Meal replacement bar
132	Milk, non-fat
133	Stuffed vegetables
134	Stalk vegetables
135	Cream, low fat
136	Dried fruit
137	Breakfast cereal, whole grain
138	Other sea-freshwater food
139	Milk, low fat, fortified
140	Yogurt, low fat
141	Herbs/spices
142	Custard, full fat
143	Rice
144	Legumes
145	Beans
146	Coconut and coconut products
147	Cheese substitute
148	Cereal flours
149	Ice cream, low fat
150	Fruit bar-sweet
151	Organ meat
152	Honey and sugar syrups
153	Yogurt, flavored or added fruit, low fat, diet
154	Pasta and noodles, whole wheat
155	Supplementary/medical beverage
156	Tomato products
157	Stock
158	Tea with milk
159	Soft drinks
160	Sugar
161	Beers, regular
162	Wine
163	Beers, lite
164	Cordials

Group Number	Food group
165	Bananas
166	Spiked soft drink
167	Fruit drinks
168	Fruit juices
169	Pome fruit
170	Cocktails
171	Electrolyte drinks (sports drinks)
172	Other fruit
173	Citrus fruit
174	Spirits
175	Stone fruit
176	Tea
177	Mixed fruit
178	Tropical fruit
179	Sugar-based desserts
180	Peas and edible-podded peas
181	Cider
182	Stone fruit, canned
183	Berry fruit
184	Leaf vegetables
185	Fruit juices, fortified
186	Wine, lite
187	Mixed fruit, canned
188	Diet sweeteners
189	Chewing gum, no sugar
190	Vegetables, wild
191	Pome fruit, canned
192	Desserts, diet
193	Cordials, diet
194	Vegetable juices
195	Other vegetables
196	Lemons and limes
197	Soft drinks, diet
198	Fruits, wild
199	Chewing gum, sugar sweetened
200	Seaweeds
201	Energy drinks, diet
202	Herbs, fresh
203	Sprouts
204	Essences
205	Water

NATISNPAS National Aboriginal and Torres Strait Islander Nutrition and Physical Activity Survey 24HR 24-hour recall

Table S2. Total amount in grams of fresh/frozen fruit items reported in the NATSINPAS and weights for calculating the energy, dietary fiber and potassium values of the composite fresh/frozen fruit FFQ item

Food item name	Total grams reported for fruit item	Proportion of total fruit intake	Energy kJ/100g of item	Weighted contribution to Energy of composite FFQ item (kJ/100g)	Fiber g/100g of item	Weighted contribution to Fiber of composite FFQ item (g/100g)	Potassium mg/100g of item	Weighted contribution to Potassium of composite FFQ item (mg/100g)
Banana, Cavendish, peeled, raw	22,826	0.26	366	97	2.4	0.6	346	92
Apple, red skin, unpeeled, raw	14,740	0.17	224	38	2.3	0.4	99	17
Orange, peeled, raw	9,960	0.12	157	18	2.6	0.3	147	17
Apple, green skin, unpeeled, raw	5,475	0.06	183	12	2.4	0.2	101	6
Grape, Thompson seedless or sultana, raw	4,802	0.06	257	14	2.3	0.1	207	12
Mandarin, peeled, raw	4,391	0.05	156	8	1.2	0.1	130	7
Melon, watermelon, peeled, raw	3,867	0.04	96	4	0.4	0.0	98	4
Pear, unpeeled, raw	3,005	0.03	219	8	3.6	0.1	110	4
Nectarine, yellow, unpeeled, raw	2,582	0.03	164	5	2.0	0.1	237	7
Plum, unpeeled, raw	2,187	0.03	146	4	2.0	0.1	153	4
Nectarine, white, peeled or unpeeled, raw	1,389	0.02	164	3	2.0	0.0	237	4
Peach, unpeeled, raw	1,341	0.02	149	2	1.9	0.0	201	3
Kiwifruit, green (Hayward), peeled, raw	1,287	0.01	187	3	3.8	0.1	259	4
Grape, raw	1,083	0.01	272	3	2.7	0.0	229	3
Grape, red globe, raw	854	0.01	294	3	3.5	0.0	270	3
Apple, red skin, peeled, raw	820	0.01	243	2	2.1	0.0	96	1
Melon, rock melon (cantaloupe), peeled, raw	759	0.01	91	1	1.0	0.0	215	2
Pear, raw	572	0.01	220	1	3.6	0.0	113	1
Peach, white, unpeeled, raw	508	0.01	141	1	1.9	0.0	196	1
Peach, yellow, unpeeled, raw	435	0.01	152	1	1.9	0.0	203	1
Pear, nashi, peeled or unpeeled, raw	380	0.00	192	1	2.1	0.0	130	1
Pear, peeled, raw	376	0.00	221	1	3.3	0.0	129	1

Food item name	Total grams reported for fruit item	Proportion of total fruit intake	Energy kJ/100g of item	Weighted contribution to Energy of composite FFQ item (kJ/100g)	Fiber g/100g of item	Weighted contribution to Fiber of composite FFQ item (g/100g)	Potassium mg/100g of item	Weighted contribution to Potassium of composite FFQ item (mg/100g)
**Apple, red delicious, unpeeled, raw**	328	0.00	230	1	2.3	0.0	102	0
**Apple, unpeeled, raw**	316	0.00	218	1	2.3	0.0	99	0
**Apple, pink lady, unpeeled, raw**	306	0.00	228	1	2.4	0.0	88	0
**Nectarine, yellow, peeled, raw**	287	0.00	170	1	1.9	0.0	242	1
**Grapefruit, peeled, raw**	279	0.00	111	0	1.7	0.0	120	0
**Melon, honey dew, peeled raw**	236	0.00	120	0	1.0	0.0	155	0
**Apple, green skin, peeled, raw**	203	0.00	207	0	2.1	0.0	104	0
**Kiwifruit, green (Hayward), unpeeled, raw**	172	0.00	182	0	4.2	0.0	273	1
**Pear, green, peeled, raw**	151	0.00	219	0	3.3	0.0	132	0
**Apricot, raw**	100	0.00	151	0	2.5	0.0	335	0
**Grape, black muscatel, raw**	85	0.00	328	0	2.7	0.0	160	0
**Cherry, raw**	56	0.00	238	0	1.5	0.0	230	0
**Total**	**86,159**	**Summed composite nutrient value:**		**Energy (kj/100g)** **236**		**Dietary Fiber (g/100g)** **2.3**		**Potassium (mg/100g)** **197**

Derivation of the Food Frequency Questionnaire (FFQ) item nutrient values in the Kaat Koort Research study

The nutrient values for the foods and beverages in the study FFQ food database were derived from the Australian Food and Nutrient Database nutrient values of the individual food items, weighted by their consumption quantity as reported in the 24HR data of the National Aboriginal and Torres Strait Islander Nutrition and Physical Activity Survey (NATSINPAS). Supplemental Table 2 presents the 35 individual items reported in the 24HR data that were combined into the study FFQ item of ‘fresh/frozen fruit’, with the total amount in grams for each item and weight for calculating the contribution of each reported 24HR item to the nutrient values of the composite FFQ item.
