# Supplementary material for: Development and Functionality of a Parsimonious Digital Food Frequency Questionnaire for a Clinical Intervention among an Indigenous Population
Source: Nutrients. 2023 Dec 4;15(23):5012. doi: 10.3390/nu15235012 (PMC10707983; doi:10.3390/nu15235012)

File S3: Screenshot of Digital FFQ with explanation of Extra item list usage

The screenshot of the digital FFQ platform shows the Bread food subgroup, with two items on the main FFQ list (1-white flour and 2-wholemeal/mixed grain bread/toast). Clicking on the 'Extra items check' button, opens the list of additional bread group items in the food database, which were reported in the NATSINPAS 24HR data or added on the advice of dietitian/community consultants), but were included in the parsimonious main FFQ food list. The list was checked and additional foods the respondent reported consuming at least once in the past month were added. Consumption information (frequency per day or month, and if consumed, number of portions) must be entered for all items on the FFQ, whether from the main list or added from extra items, in order to complete the assessment.

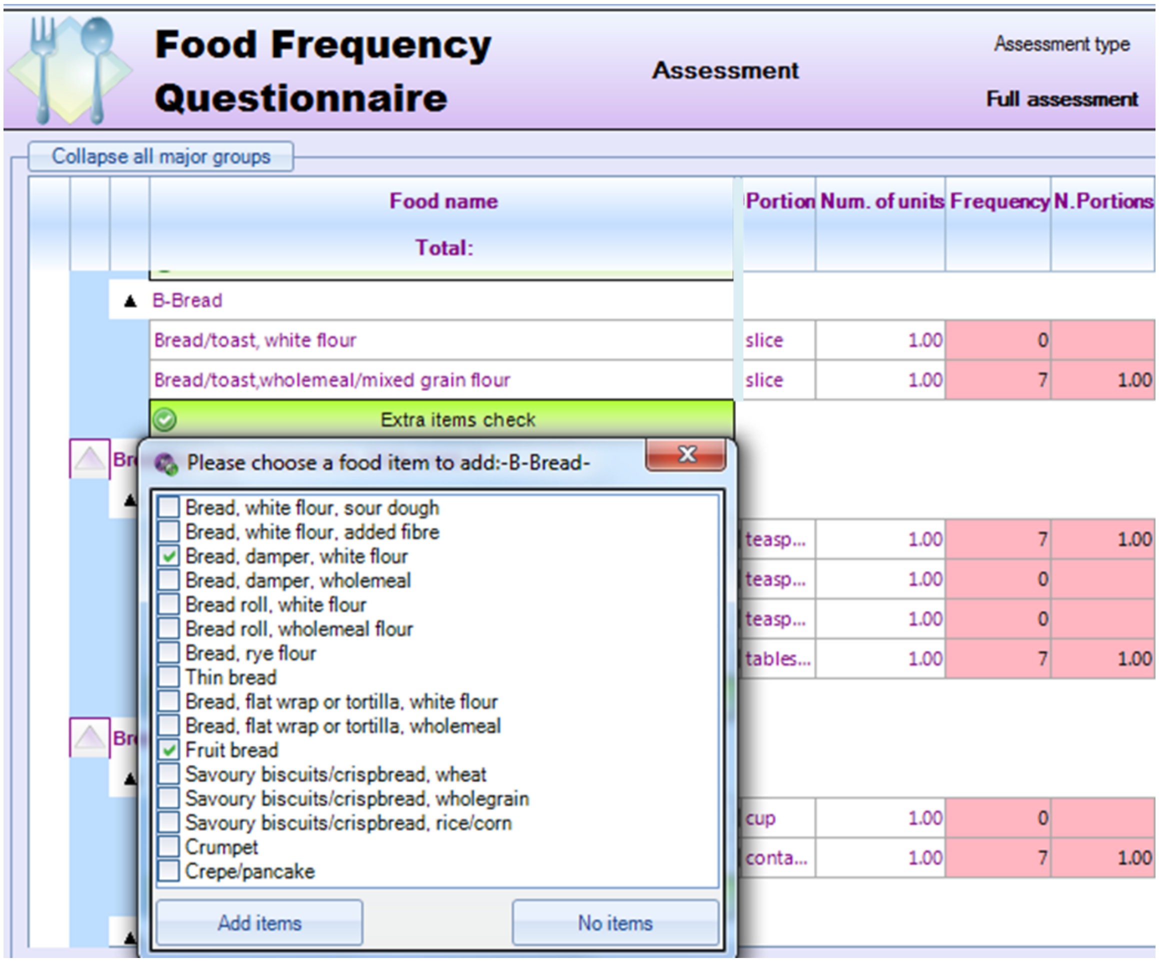

Supplement: Supplementary file 1 [file nutrients-15-05012-s001.zip › Supplemental File S3-Screenshot of Digital FFQ with explanation of Extra item list usage.pdf]
