# Supplementary material for: Development and Functionality of a Parsimonious Digital Food Frequency Questionnaire for a Clinical Intervention among an Indigenous Population
Source: Nutrients. 2023 Dec 4;15(23):5012. doi: 10.3390/nu15235012 (PMC10707983; doi:10.3390/nu15235012)
Supplement: Supplementary file 1 [file nutrients-15-05012-s001.zip › Supplemental File S4. Detailed 24HR contributors to kJ.pdf]

Supplemental Table S4. Detailed list 24HR food and beverage group contributors to kJ and other nutrients of interest

Contributors	Percent contribution to total energy intake
Contributors to Energy	
Breads, white,fort	6.45
Beef-lamb-pork	4.70
Soft drinks	3.75
Processed meat	3.47
Hot potato chips	3.40
Milk, full fat	3.13
Mixed poultry dishes	3.08
Savoury pasta/rice dishes	3.06
Cakes	2.71
Poultry	2.69
Burgers	2.63
Savoury pastry	2.52
Sugar	2.18
Mixed red-meat dishes	2.17
Beers, regular	2.01
Pizza	1.79
Wine	1.66
Cheese, ripened, hi fat	1.61
Beers, lite	1.54
Potatoes	1.49
Fin fish, fried	1.32
Breads, whole grain,fort	1.27
Breads, mixed grain,fort	1.21
Ice cream, full fat	1.19
Breakfast cereal, whole grain, fort, lo sugar	1.17
Cordials	1.15
Chocolate, filled	1.07
Rice	1.02
Margarine	0.97
Eggs	0.94
Milk drinks, full fat	0.92
Bananas	0.92
Butter	0.89
Breakfast cereal, muesli	0.83
Sweet biscuits, filled	0.82
Spiked soft drink	0.79
Chocolate, plain	0.78
Milk, lo fat	0.78
Fruit drinks	0.75
Sandwiches	0.74
Potato crisps	0.74
Porridge	0.72

Supplemental Table S4. Detailed list 24HR food and beverage group contributors to kJ and other nutrients of interest

Contributors	Percent contribution to total energy intake
Pasta and noodles	0.69
Fruit juices	0.68
Pome fruit	0.66
Egg dishes, savoury	0.60
Coffee with milk	0.60
Subtotal	80.25
Additional Contributors to Protein	
Fin fish, fresh	0.34
Fin fish, preserved	0.33
Sports/protein beverage	0.43
Subtotal	1.10
Additional Contributors to Total Fat*	
Sweet biscuits, filled	0.82
Dairy desserts	0.47
Other nuts	0.53
Salad dressing, full fat	0.53
Squash	0.40
Salads, vegetable based	0.47
Subtotal	3.21
Additional Contributors to Carbohydrates	
Electrolyte drinks (sports drinks)	0.56
Gravies	0.58
Honey and sugar syrups	0.29
Candies, sugar sweetened	0.36
Cocktails	0.59
Subtotal	2.38
Additional Contributors to Fibre, Sodum, Potassium, Magnesium	
Citrus fruit	0.26
Stone fruit	0.17
Other fruit	0.29
Soup with meat, homemade	0.34
Brassica vegetables	0.07
Carrots	0.15
Other root vegetables	0.19
Peas and edible-podded peas	0.10
Tomato	0.15
Squash	0.40
Sweetcorn	0.18
Mixed vegetables	0.30
Salads, vegetable based	0.47
Legume products	0.20
Water	0.00
Taco-tortilla	0.34

Supplemental Table S4. Detailed list 24HR food and beverage group contributors to kJ and other nutrients of interest

**Contributors**	**Percent contribution to total energy intake**
Doughnut/crepe/pancake	0.47
Yeast	0.08
Fortified beverage	0.29
Subtotal	4.45
**TOTAL**	**91.40**

*Includes fat from preparation or serve methods (e.g., stir-fried; additions of cheese, nuts, dressings)
