# Supplementary material for: Development and Functionality of a Parsimonious Digital Food Frequency Questionnaire for a Clinical Intervention among an Indigenous Population
Source: Nutrients. 2023 Dec 4;15(23):5012. doi: 10.3390/nu15235012 (PMC10707983; doi:10.3390/nu15235012)
Supplement: Supplementary file 1 [file nutrients-15-05012-s001.zip › Supplemental File S5. Contributions of the main and extra I-ACE FFQ items by sex.pdf]

Table S5. Contributions of the main and 'extra'[1] I-ACE FFQ items to (A) the total intake of energy, and selected nutrients and (B) food groups reported by initial Kaat Koort participants by sex (n=60)

	Percent contribution to total reported intake			
	Women (n=42)		Men (n=18)	
Nutrient or food group	Main items	Extra items	Main items	Extra items
**A. Energy and nutrients**				
Energy (kJ)	68.8	31.2	70.6	29.4
Protein (g)	64.3	35.7	68.8	31.2
Total fat (g)	64.9	35.1	66.9	33.1
Fibre (g)	81.5	18.5	79.8	20.2
Carbohydrates (g)	73.7	26.3	73.4	26.6
Total sugar (g)	74.5	25.5	76.0	24.0
Free sugar (g)	77.5	22.5	76.7	23.3
Calcium (mg)	62.4	37.6	67.8	32.2
Sodium (mg)	69.5	30.5	66.1	33.9
Magnesium (mg)	67.1	32.9	70.0	30.0
Potassium (mg)	70.6	29.4	73.8	26.2
**B. Food groups (serves)**				
Total grains	78.9	21.1	77.8	22.2
Whole grains	86.5	13.5	80.4	19.6
Total Vegetables	91.5	8.5	86.4	13.6
Fruit	93.8	6.2	89.0	11.0
Total dairy	51.1	48.9	62.3	37.7
Low fat dairy	42.0	58.0	62.3	37.7
Meats and alternative protein sources[2]	47.5	52.5	64.2	35.8
Fast/fried food	65.8	34.2	59.8	40.2
Processed/salty food	68.5	31.5	49.9	50.1
Alcoholic drinks	68.0	32.0	79.7	20.3

I-ACE Interactive Lifestyle Assessment, Counseling and Education, FFQ food frequency questionnaire

[1]Items not on the main food list, that were chosen by participants who consumed them from the "extra items" lists

[2] Excluding dairy (which are in the dairy group) and legumes (which are in the vegetables group)
