# Supplementary material for: Development and Functionality of a Parsimonious Digital Food Frequency Questionnaire for a Clinical Intervention among an Indigenous Population
Source: Nutrients. 2023 Dec 4;15(23):5012. doi: 10.3390/nu15235012 (PMC10707983; doi:10.3390/nu15235012)
Supplement: Supplementary file 1 [file nutrients-15-05012-s001.zip › Supplemental File S6. Offline iACE FFQ-r1.pdf]

Supplemental File S6. Offline Kaat Koort Food Frequency Questionnaire

TEMPLATE: Fill in 'Frequency'* & 'Number of portions' for all items consumed **at least once a month**.
Ask about Extra items; if yes add item name, portion, frequency and # portions.
[§]Item repeated in 1 other meal. [§§]Item repeated in 2 other meals.

Participant ID: ________ Visit #: ___ Date: __________<br>**FFQ items**	*Portion size*	*# units in portion*	Frequency*	Number of portions
**Breakfast/Brunch**				
**Grains-Starches**				
Breakfast cereal, refined (corn, oats, rice)[§]	*cup*	*1*		
Breakfast cereal, whole grain (Weetbix, All Bran)	*cup*	*1*		
Porridge, rolled oats, cooked	*cup*	*1*		
Sugar, white (for cereal)	*teaspoon*	*1*		
**Extra items:** (e.g., sugar-coated cereal, muesli bars)				
		1		
		1		
**Bread**				
Bread/toast, white flour[§§]	*slice*	*1*		
Bread/toast,wholemeal/mixed grain flour[§§]	*slice*	*1*		
**Extra items:** (e.g., damper, crumpets, savoury biscuits)				
		1		
		1		
**Spreads-Sauces**				
Butter, plain, salted[§§]	*teaspoon*	*1*		
Margarine, regular[§§]	*teaspoon*	*1*		
Vegemite/Promote[§§]	*teaspoon*	*1*		
Honey	*teaspoon*	*1*		
**Extra items:** (e.g., peanut butter, reduced-salt margarine, jam)				
		1		
		1		
		1		

*Frequency:
If consumed at least once a week, provide exact number of days per week (1 to 7).
If consumed at least once a month but less than once a week, provide code for times per month (M1, M2, M3).

TEMPLATE: Fill in 'Frequency'* & 'Number of portions' for all items consumed **at least once a month**.
Ask about Extra items; if yes add item name, portion, frequency and # portions.
§Item repeated in 1 other meal. §§Item repeated in 2 other meals.

Participant ID: ________ Visit #: ___ Date: __________ **FFQ items**	*Portion size*	*# units in portion*	**Frequency***	**Number of portions**
**Dairy products**				
Milk,full cream (for cereal)	*cup*	*1*		
Yoghurt, regular fat, flavored	*container*	*1*		
**Extra items:** (e.g., Hilo/skimmed milk, milk powder, plain yoghurt, cheese)				
		1		
		1		
**Eggs**				
Eggs, fried/scrambled§	*unit*	*1*		
Savory egg dishes (omelette/scrambled, + additions)	*serve*	*1*		
**Extra items:** (e.g., hard-boiled/poached eggs)				
		1		
		1		
**Meat (alone, or in stews/sauces/casseroles/curry/sandwiches)**				
Bacon	*slice*	*1*		
Sausage§	*unit*	*1*		
**Extra items:** (e.g., tinned or fresh fish, seafood, ham)				
		1		
		1		
**Vegetables (alone, or in mixed dishes)**				
Potato, sweet potato, sweetcorn§§	*Exchange equivalent*	*1*		
Baked beans§§	*cup*	*1*		
**Extra items:** (e.g., fresh vegetables/salads)				
		1		
		1		

*Frequency:
If consumed at least once a week, provide exact number of days per week (1 to 7).
If consumed at least once a month but less than once a week, provide code for times per month (M1, M2, M3).

TEMPLATE: Fill in 'Frequency'* & 'Number of portions' for all items consumed **at least once a month**.
Ask about Extra items; if yes add item name, portion, frequency and # portions.
§Item repeated in 1 other meal. §§Item repeated in 2 other meals.

Participant ID: ________ Visit #: ___ Date: __________ FFQ items	*Portion size*	*# units in portion*	**Frequency***	**Number of portions**
**Fast foods**				
Muffin, English style, with sausage or bacon/egg/cheese	*unit*	*1*		
**Extra items:** (e.g., pastie/sausage roll, hot potato chips)				
		1		
		1		
**Midday and evening meals**				
**Meat (alone, or in stews/sauces/casseroles/curry/sandwiches/meatballs)**				
Chicken/poultry, with skin	*serve*	*1*		
Beef/lamb/pork, visible fat/mince	*serve*	*1*		
Sausage§	*unit*	*1*		
Kangaroo/rabbit/wild game	*serve*	*1*		
Cooked fish	*serve*	*1*		
Tinned tuna and other tinned fish	*can*	*1*		
**Extra items:** (e.g., chicken without skin, lean beef, ham/luncheon meats, seafood)				
		1		
		1		
**Vegetables (alone or in stews/casseroles/sauces/curry/stirfry)**				
Potato, sweet potato, sweetcorn§§	*Exchange equivalent*	*1*		
All other vegetables/salads§	*cup*	*0.5*		
Baked beans§§	*cup*	*1*		
**Extra items:** (e.g., homemade hot potato chips, potato/rice/pasta salad, other legumes)				

*Frequency:
If consumed at least once a week, provide exact number of days per week (1 to 7).
If consumed at least once a month but less than once a week, provide code for times per month (M1, M2, M3).

TEMPLATE: Fill in 'Frequency'* & 'Number of portions' for all items consumed **at least once a month**.
Ask about Extra items; if yes add item name, portion, frequency and # portions.
§Item repeated in 1 other meal. §§Item repeated in 2 other meals.

Participant ID: ________ Visit #: ___ Date: __________ **FFQ items**	*Portion size*	*# units in portion*	**Frequency***	**Number of portions**
		1		
		1		
**Bread**				
Bread/toast, white flour§§	*slice*	*1*		
Bread/toast,wholemeal/mixed grain flour§§	*slice*	*1*		
**Extra items:** (e.g., savoury biscuits, bread rolls, flat wraps/tortillas, damper)				
		1		
		1		
**Grains-Starches**				
Rice, white (incl. in mixed dishes)	*heaped serving spoon*	*1*		
Pasta, noodles (incl. in mixed dishes)	*heaped serving spoon*	*1*		
**Extra items:** (e.g., brown rice, wholegrain noodles/pasta, porridge, muesli bars, breakfast cereal)				
		1		
		1		
**Spreads-Dips**				
Butter, plain, salted§§	*teaspoon*	*1*		
Margarine, regular§§	*teaspoon*	*1*		
Tomato sauce (ketchup)	*tablespoon*	*1*		
Mayonaise, regular fat	*tablespoon*	*1*		
Vegemite/Promote§§	*teaspoon*	*1*		
**Extra items:** (e.g., avocado, peanut butter, reduced salt butter/margarine)				
		1		
		1		

*Frequency:
If consumed at least once a week, provide exact number of days per week (1 to 7).
If consumed at least once a month but less than once a week, provide code for times per month (M1, M2, M3).

TEMPLATE: Fill in 'Frequency'* & 'Number of portions' for all items consumed **at least once a month**.
Ask about Extra items; if yes add item name, portion, frequency and # portions.
§Item repeated in 1 other meal. §§Item repeated in 2 other meals.

Participant ID: ________ Visit #: ___ Date: __________<br>**FFQ items**	***Portion size***	***# units in portion***	**Frequency***	**Number of portions**
**Sauces (on meat, stew/casseroles, pasta/noodles, rice, vegetables)**				
Gravy	*tablespoon*	*1*		
Asian marinade/soy sauce	*tablespoon*	*1*		
Tomato sauce for pasta bolognese, salsa, tacos	*cup*	*0.5*		
**Extra items:** (e.g., cream-based sauces, salad dressings, barbeque sauce, chutney/relish)				
		1		
		1		
**Dairy**				
Cheese (chedder, any other hard yellow cheese)§	*slice*	*1*		
**Extra items:** (e.g., yoghurt, soft/spread cheeses, weight loss milk shakes, milk for cereal)				
		1		
		1		
**Eggs**				
Eggs, fried/scrambled§	*unit*	*1*		
**Extra items:** (e.g., hard-boiled/poached eggs, omelette/scrambled eggs)				
		1		
		1		
**Fast foods**				
Pizza, with meat	*slice*	*1*		
Hot dog, bread roll	*unit*	*1*		
Hamburger, bread roll, beef patty§	*unit*	*1*		
Hot potato chips/fries/wedges, fast food/take away§	*serve*	*1*		

*Frequency:
If consumed at least once a week, provide exact number of days per week (1 to 7).
If consumed at least once a month but less than once a week, provide code for times per month (M1, M2, M3).

TEMPLATE: Fill in 'Frequency'* & 'Number of portions' for all items consumed **at least once a month**.
Ask about Extra items; if yes add item name, portion, frequency and # portions.
§Item repeated in 1 other meal. §§Item repeated in 2 other meals.

Participant ID: ________ Visit #: ___ Date: __________ FFQ items	Portion size	# units in portion	Frequency*	Number of portions
**Extra items:** (e.g., pastie/pie, grilled chicken wrap/burger, crispy/coated chicken, fried fish, toasted cheese sandwich)				
		1		
		1		
**Deep-fat Frying (meat/fish/vegetables)**				
Oil for deep-fat frying	tablespoon	1		
**Between meal snacks / desserts**				
**Bread**				
Bread/toast, white flour§§	slice	1		
Bread/toast,wholemeal/mixed grain flour§§	slice	1		
**Extra items:** (e.g., savoury biscuits, damper, crumpet, fruit bread)				
		1		
		1		
**Grains-Starches**				
Breakfast cereal, refined grains (corn, oat, rice)§	cup	1		
**Extra items:** (e.g., muesli/protein bars, breakfast cereals/porridge, noodles/pasta)				
		1		
		1		
**Spreads-Sauces**				
Butter, plain, salted§§	teaspoon	1		
Margarine, regular§§	teaspoon	1		

*Frequency:
If consumed at least once a week, provide exact number of days per week (1 to 7).
If consumed at least once a month but less than once a week, provide code for times per month (M1, M2, M3).

TEMPLATE: Fill in 'Frequency'* & 'Number of portions' for all items consumed **at least once a month**.
Ask about Extra items; if yes add item name, portion, frequency and # portions.
§Item repeated in 1 other meal. §§Item repeated in 2 other meals.

Participant ID: ________ Visit #: ___ Date: __________ **FFQ items**	*Portion size*	*# units in portion*	**Frequency***	**Number of portions**
Vegemite/Promote§§	*teaspoon*	*1*		
**Extra items:** (e.g., avocado, chutney/relish, peanut butter, jam)				
		1		
		1		
**Dairy**				
Cheese (chedder, any hard yellow cheese)§	*slice*	*1*		
**Extra items:** (e.g., yoghurt, soft/spread cheeses, cream)				
		1		
		1		
**Eggs**				
Eggs, hard-boiled/poached	*unit*	*1*		
**Extra items:** (e.g., fried/scrambled eggs, savoury egg dishes)				
		1		
		1		
**Meat**				
Sausage§§	*unit*	*1*		
Spam/Polony/processed luncheon meat	*slice*	*1*		
**Extra items:** (e.g., tinned fish, ham/luncheon meats, wild game, chicken, beef/lamb/pork)				
		1		
		1		
**Vegetables**				
Potato, sweet potato, sweetcorn§§	*Exchange equivalent*	*1*		
All other vegetables/salads§	*cup*	*0.5*		

*Frequency:
If consumed at least once a week, provide exact number of days per week (1 to 7).
If consumed at least once a month but less than once a week, provide code for times per month (M1, M2, M3).

TEMPLATE: Fill in 'Frequency'* & 'Number of portions' for all items consumed **at least once a month**.
Ask about Extra items; if yes add item name, portion, frequency and # portions.
[§]Item repeated in 1 other meal. [§§]Item repeated in 2 other meals.

Participant ID: ________ Visit #: ___ Date: __________ FFQ items	Portion size	# units in portion	Frequency*	Number of portions
Baked beans[§§]	cup	1		
**Extra items:** (e.g., potato/rice/pasta salad, other legumes)				
		1		
		1		
**Fast foods**				
Hamburger, bread roll, beef patty[§]	unit	1		
Hot potato chips/fries/wedges, fast food/take away[§]	serve	1		
**Extra items:** (e.g., grilled chicken salad/sandwich, pizza, pastie/sausage roll, crispy chicken burger, toasted cheese sandwich)				
		1		
		1		
**Fruit**				
Fresh/frozen fruit	serve	1		
**Extra items:** (e.g., canned fruit, dried fruit)				
		1		
		1		
**Desserts (cake, biscuits)**				
Biscuit, sweet, with filling/icing/ additions	unit	1		
Cake or cupcake, iced/filled	slice/unit	1		
**Extra items:** (e.g., banana bread, pudding, doughnut/ croissant, plain biscuits, cheesecake)				
		1		
		1		
**Lollies and ice cream**				
Ice cream, regular fat	ball/scoop	1		

*Frequency:
If consumed at least once a week, provide exact number of days per week (1 to 7).
If consumed at least once a month but less than once a week, provide code for times per month (M1, M2, M3).

TEMPLATE: Fill in 'Frequency'* & 'Number of portions' for all items consumed **at least once a month**.
Ask about Extra items; if yes add item name, portion, frequency and # portions.
§Item repeated in 1 other meal. §§Item repeated in 2 other meals.

Participant ID: ________ Visit #: ___ Date: __________ FFQ items	*Portion size*	*# units in portion*	**Frequency***	**Number of portions**
Chocolate, milk/dark/white	*unit*	*4*		
Lollies, all varieties	*unit*	*1*		
**Extra items:** (e.g., chocolate-coated bars, toppings. diet [lo-fat, lo-sugar] ice cream,)				
		1		
		1		
**Nuts**				
Nuts, all types, roasted, salted	*handful*	*1*		
**Extra items:** (e.g., unsalted nuts)				
		1		
		1		
**Salty snacks**				
Potato or veggie crisps/chips	*packet*	*1*		
**Extra items:** (e.g., twisties/cheezels, popcorn, pretzels, olives)				
		1		
		1		

**Drinks**				
**Hot drinks**				
Tea, regular, black	*cup*	*1*		
Coffee, instant or ground (prepared with water)	*cup*	*1*		
Sugar, white (for tea/coffee)	*teaspoon*	*1*		
**Extra items:** (e.g., cappuccino/latte, herbal tea, coffee mix-prepared)				
		1		

*Frequency:
If consumed at least once a week, provide exact number of days per week (1 to 7).
If consumed at least once a month but less than once a week, provide code for times per month (M1, M2, M3).

TEMPLATE: Fill in 'Frequency'* & 'Number of portions' for all items consumed **at least once a month**.
Ask about Extra items; if yes add item name, portion, frequency and # portions.
§Item repeated in 1 other meal. §§Item repeated in 2 other meals.

**Participant ID:** ________ **Visit #:** ___ **Date:** __________<br>**FFQ items**	*Portion size*	*# units in portion*	**Frequency***	**Number of portions**
		1		
**Milk drinks**				
Milk, full cream (incl. added to tea/coffee)	*cup*	*1*		
**Extra items:** (e.g., Hilo milk, flavoured milk, shakes, dairy smoothies)				
		1		
		1		
**Cold drinks**				
Water (tap, bottled, mineral, sparkling)	*cup*	*1*		
100% juice, natural	*cup*	*1*		
Soft drink/fruit drink/cordial/slushie, regular	*cup*	*1*		
**Extra items:** (e.g., energy/sports drinks, diet drinks, non-dairy milks)				
		1		
		1		
**Alcoholic drinks**				
Beer, regular	*can*	*1*		
Wine, red/white	*wine glass*	*1*		
Mixed drinks/cocktails (rum/whisky/scotch and cola)	*can*	*1*		
**Extra items:** (e.g., light beer, spirits)				
		1		
		1		

*Frequency:
If consumed at least once a week, provide exact number of days per week (1 to 7).
If consumed at least once a month but less than once a week, provide code for times per month (M1, M2, M3).

TEMPLATE: Fill in 'Frequency'* & 'Number of portions' for all items consumed **at least once a month**.
Ask about Extra items; if yes add item name, portion, frequency and # portions.
§Item repeated in 1 other meal. §§Item repeated in 2 other meals.

**Participant ID:** ________ **Visit #:** ___ **Date:** __________<br>**FFQ items**	***Portion size***	***# units in portion***	**Frequency***	**Number of portions**
**Supplements**				
Multivitamin and/or multimineral	*unit*	*1*		
**Extra items:** (e.g., Vitamin D, protein/amino acid supplement, other mineral supplements)				
		1		
		1		

*Frequency:
If consumed at least once a week, provide exact number of days per week (1 to 7).
If consumed at least once a month but less than once a week, provide code for times per month (M1, M2, M3).
